# The Dynamic Distribution of *Wolbachia* and *Rickettsia* in AsiaII1 *Bemisia tabaci*

**DOI:** 10.3390/insects14040401

**Published:** 2023-04-21

**Authors:** Ning Lv, Jing Peng, Zi-Qi He, Qin Wen, Zheng-Qin Su, Shaukat Ali, Chang-Zhong Liu, Bao-Li Qiu

**Affiliations:** 1 Biocontrol Engineering Laboratory of Crop Diseases and Pests of Gansu Province, College of Plant Protection, Gansu Agricultural University, Lanzhou 730070, China; 2 Key Laboratory of Bio-Pesticide Innovation and Application of Guangdong Province, South China Agricultural University, Guangzhou 510642, China; 3 Engineering Research Center of Biocontrol, Ministry of Education, Guangzhou 510642, China; 4 Chongqing Key Laboratory of Vector Insects, College of Life Sciences, Chongqing Normal University, Chongqing 401331, China

**Keywords:** *Bemisia tabaci*, *Wolbachia*, *Rickettsia*, spatial and temporal distribution, endosymbiont bacteria

## Abstract

**Simple Summary:**

Bacterial endosymbionts play important roles in the life history of herbivorous insects, including supplying nutrients allowing exploitation of unbalanced diets, increasing the survivorship and fecundity of hosts, protecting hosts against entomopathogenic fungi and parasitoid wasps, ameliorating the detrimental effects of heat, and broadening the range of suitable food plants. The important function of bacterial endosymbionts is that they can manipulate the reproduction of their hosts. However, all functions of endosymbionts must rely on vertical transmission to spread within the population, such as *Wolbachia*, *Rickettsia*, and *Cardinium*. So, we studied the spatial and temporal distribution of *Wolbachia* and *Rickettsia* in Asia II 1 *Bemisia tabaci.* The results showed that the titers of *Wolbachia* and *Rickettsia* in the 3–120 h old eggs changed in a “w” pattern, and the location of *Wolbachia* and *Rickettsia* in the egg changed from egg stalk to egg base, and then from the egg base to egg posterior, and finally back to the middle of egg in a *Rickettsia* and *Wolbachia* coinfected whitefly host. Our study helps to explain the vertical transmission mechanism of bacterial endosymbionts and the distribution of bacterial endosymbionts in different tissues in the host.

**Abstract:**

*Wolbachia* and *Rickettsia* are bacterial endosymbionts that can induce a number of reproductive abnormalities in their arthropod hosts. We screened and established the co-infection of *Wolbachia* and *Rickettsia* in *Bemisia tabaci* and compared the spatial and temporal distribution of *Wolbachia* and *Rickettsia* in eggs (3–120 h after spawning), nymphs, and adults of *B. tabaci* by qPCR quantification and fluorescent in situ hybridization (FISH). The results show that the titer of *Wolbachia* and *Rickettsia* in the 3–120 h old eggs showed a “w” patterned fluctuation, while the titers of *Wolbachia* and *Rickettsia* had a “descending–ascending descending–ascending” change process. The titers of *Rickettsia* and *Wolbachia* nymphal and the adult life stages of Asia II1 *B. tabaci* generally increased with the development of whiteflies. However, the location of *Wolbachia* and *Rickettsia* in the egg changed from egg stalk to egg base, and then from egg base to egg posterior, and finally back to the middle of the egg. These results will provide basic information on the quantity and localization of *Wolbachia* and *Rickettsia* within different life stages of *B. tabaci.* These findings help to understand the dynamics of the vertical transmission of symbiotic bacteria.

## 1. Introduction

The symbiotic relationship between insects and endosymbionts is widely prevalent [1]. Obligate endosymbionts are critical determinants of host physiology by complementing the incomplete or absent eukaryotic metabolic pathways required for the synthesis of essential nutrients with limited availability in the host diets [2]. Plant-sap-sucking insects are always dependent on their primary prokaryotic endosymbionts for survival, such as *Buchnera* in aphids and *Carsonella* in psyllids [3,4]. Additionally, these host insects may have one or more facultative endosymbionts, which are not necessary for the host’s survival or reproduction, but they can have important effects on the biology and ecology of the hosts, including physiology, evolution, reproduction, immune homeostasis, and defense [5,6,7]. Intracellular bacteria of insect hosts are vertically transmitted in different ways. Bacteria are released from the bacteriocytes and transferred to the ovary in most insects, but bacteriocytes migrate to each egg, and bacteria are released from bacteriocytes in whiteflies [8]. The vertical transmission mechanism of symbiotic bacteria can reshape bacteriocytes during the developmental transition from nymph to adulthood, including the loss of cell–cell adhesion, high division rates to constant cell size, and the onset of cell mobility, so that the bacteriocytes can crawl to the ovary [9].

*Bemisia tabaci* (Gennadius, s1889) (Hemiptera: Aleyrodidae) is a complex of cryptic species, among which at least 40 cryptic species are morphologically indistinguishable but biologically different, and has become one of the most economically important crop pests worldwide [10,11]. The *B. tabaci* species complex shares a long-term and intimate association with only one obligate bacterial symbiont (*Portiera*) and facultative bacterial endosymbionts (belonging to seven genera *Arsenophonus*, *Cardinium*, *Hamiltonella*, *Hemipteriphilus*, *Wolbachia*, *Fritschea*, and *Rickettsia*) [12]. *Wolbachia* and *Rickettsia* are the most studied endosymbionts. Both of them can affect some aspects of host fitness, such as weight, fecundity, longevity, and defense [13,14,15]. Furthermore, *Wolbachia* and *Rickettsia* are the master manipulators of arthropod reproduction that can induce a number of reproductive abnormalities, such as male killing, feminization, parthenogenesis, and cytoplasmic incompatibility (CI), thus increasing the spread of *Wolbachia* in host populations [16,17]. CI has attracted much attention due to its application in the biological control of insect pests [16,17,18,19,20]. The utilization of *Wolbachia* or *Rickettsia* as a pest control agent requires generating novel *Wolbachia* or *Rickettsia*–host interactions by transinfection or microinjection of *Wolbachia* or *Rickettsia* strains into different insect pests [21]. Although there are a large number of studies on the effects of *Wolbachia* or *Rickettsia* on host fitness and behavior and their potential application as biological control agents [22], there is a lack of detailed information on the behavior of bacteria within different tissues during the host’s life cycle.

In this study, the spread of *Wolbachia* and *Rickettsia* spread within different life stages of *B. tabaci* was detected under laboratory conditions. We compared the spatial and temporal distribution of *Wolbachia* and *Rickettsia* in eggs (3–120 h after egg laying), nymphs, and adults of *B. tabaci* using qPCR quantification and fluorescent in situ hybridization (FISH). These results will further enhance our understanding of the vertical transmission of symbiotic bacteria in insects. This study will also provide basic dynamic change pattern on the quantity and localization of *Wolbachia* and *Rickettsia* within different life stages of *B. tabaci* and will serve as a reference for the selection of the optimal time for the horizontal transfer of symbiotic bacteria within the eggs of different whiteflies by microinjection techniques, to study the interactions between the same symbiotic bacteria and different whitefly cryptic species or transfection of symbiotic bacteria inducing cytoplasmic incompatibility for pest control by microinjection technique.

## 2. Materials and Methods

### 2.1. Host Plant and Whitefly Cultures

The cotton plant *Gossypium hirsutum* Linnaeus 1763. (var. Lumianyan no. 32) was used to rear *B. tabaci* Asia II 1. Cultivate cotton seedlings according to Li et al. [23]. Healthy plants at the six to eight expanded-leaf stage were used in experiments. *B. tabaci* Asia II 1 individuals were collected from the soybean plants, *Glycine max* (Linn.) Merr in Guangzhou in 2017 and reared on the cotton plants at 26.0 ± 0.5 °C and 70–80% relative humidity under a 14:10 h (L:D) photoperiod in the laboratory at South China Agricultural University. The species and infection rate of symbiotic bacteria in the original population of *B. tabaci* were determined by PCR detection, and the experimental population line was screened and established based on the original laboratory line using the “single-pair” and PCR detection method (newly emerged single pair of whiteflies (one female and one male) were released into a leaf cage, which was attached onto a clean cotton leaf to allow egg laying to establish the population), and a laboratory line that was coinfected with *Rickettsia* and *Wolbachia* (I_WR_).

### 2.2. PCR Detection of Rickettsia and Wolbachia in Asia II1 Whitefly

On the basis of clarifying the type and infection rate of secondary endosymbionts, newly emerged adults were randomly selected in the original population was measured by PCR with special primers of *16S rRNA*, *23S rRNA*, and *wsp* genes of endosymbionts (Supplementary Table S1). Results revealed that the original laboratory line of Asia II1 whitefly was infected with *Rickettsia* and *Wolbachia*, but the infection percentages of *Rickettsia* and *Wolbachia* in whitefly adults were 100% and 83.3% (Supplementary File 1). So, the experimental line with co-infection of *Rickettsia* and *Wolbachia* was established using the “single-pair screen” and PCR detection method described by Liu et al. [24]. Adult individuals were randomly selected for the PCR detection of secondary endosymbionts *Rickettsia* and *Wolbachia*. Specific primers and cycling conditions are shown in Table S1. Total genomic DNA of individual whitefly was extracted according to Ahmed et al. [25]. PCRs were performed in a 25 μL volume containing 1 μL of the template DNA lysate, 2.5 mM MgCl_2_, 200 mM for each dNTP, 1 μM of each primer, and 1 unit of DNA Taq polymerase (Invitrogen, Guangzhou, China). A 3 μL volume of the PCR product was visualized on 1% agarose gel containing GoldView colorant (Invitrogen, Guangzhou, China) and then photographed. When bands with the expected size were visible on the gels, the remaining 17 μL of PCR product was sent for sequencing in Sangon Biotech (Shanghai) Co., Ltd. Portiera aleyrodidarum DNA was used as a positive control and ddH_2_O was used as a negative control to eliminate potential confounding variables and judge the quality of extracted DNA.

### 2.3. Infection Monitoring and Maternal Transmission Efficiency of Rickettsia and Wolbachia in Laboratory Lines

The co-infection status of *Rickettsia* and *Wolbachia* in the I_WR_ line, which included both females and males, was monitored prior to and during the experiments using polymerase chain reaction (PCR) using *Rickettsia*-specific *16S rRNA* and *Wolbachia*-specific *wsp* primers as described previously [26,27]. A single experiment to detect the endosymbiont of whitefly was considered as one repeat, and the experiment was repeated 60 times. The infection status of *Rickettsia* and *Wolbachia* was monitored for three generations. The maternal transmission efficiency of *Rickettsia* and *Wolbachia* was studied according to the methods of Nguyen [28]. Ten mothers were arbitrarily selected and tested to determine if they undergo arrhenotokous or amphigenetic reproduction. Then, the maternal transmission efficiency of *Rickettsia* and *Wolbachia* was recorded in the arrhenotokous parthenogenetic progeny of I_WR_ virgin females and the amphigenetic progeny of I_WR_ virgin females and I_WR_ virgin males. The infection status of *Rickettsia* and *Wolbachia* was tested with PCR using *Rickettsia*-specific *16S rRNA* and *Wolbachia*-specific *wsp* primers (Supplementary Table S1) to measure the maternal transmission efficiency of *Rickettsia* and *Wolbachia*, and 10 male or female progenies of each mother were examined for a total of 100 progeny individuals per setup.

### 2.4. Quantitative Detection of Rickettsia and Wolbachia

To collect eggs, nymphs, and newly emerged males and females of Asia II1 for quantitative detection of endosymbionts, 500 females and males of I_WR_ lines were isolated with leaf cages on cotton leaves. The tested insects were allowed to oviposit for 3 h, followed by their removal from the cages. Eggs were collected at 3, 6, 9, 12, 18, 24, 36, 48, 72, 96, and 120 h post adult removal. Newly emerged nymphs of each instar (from first to fourth instar) samples were collected. Newly emerged adults were collected and separated with dactylethrae, and their sex was examined under a microscope. DNA samples were extracted using a TIANamp Genomic DNA kit (Tiangen, Beijing, China). Quantitative real-time PCR (qPCR) was used to detect the titer of *Rickettsia* and *Wolbachia* in the line of *B. tabaci* Asia II1 following the protocol of Ghanim and Kontsedalov [29]. The qPCR was performed in a 10 μL reaction system, including 5 μL SYBR Premix (ThunderbirdTM, Osaka, Japan), 10 μmol of each primer, 2 μL extracted DNA, and 1 μL ddH_2_O (Total 10 μL). The procedure was 5 min activation at 95 °C, then 45 cycles of 20 s at 95 °C, 30 s at 60 °C, and 45 s at 72 °C in a quantitative real-time PCR machine (CFX-96, Bio-Rad Co. Ltd.). Melting ramp included 30 s at 50 °C to 98 °C, rising 1 °C at each step, and waiting 5 s after each step. The primers of *gltA* and *coxA* genes were used as described previously [30,31]. A β-actin whitefly gene was selected as an internal control for data normalization and quantification [29]. Thirty sets of eggs, thirty nymphs of each instar, and thirty pairs of newly emerged males and females of the I_WR_ line quantified the titer of *Rickettsia* and *Wolbachia* as one repeat, and the experiment was repeated three times. Three technical replicates were performed for each biological replicate.

### 2.5. Fluorescence In Situ Hybridization Microscopy of Rickettsia and Wolbachia in Asia II1 Whiteflies

Eggs, nymphs, and adults of Asia II1 were collected for fluorescence in situ hybridization visualization of endosymbionts. Localizations of *Wolbachia* and *Rickettsia* in the eggs, nymphs, and adults of whiteflies was studied by FISH using the previously described protocol [31]. Whitefly samples were fixed overnight in Carnoy’s solution (chloroform–ethanol–acetic acid 6:3:1) at 4 °C. The samples were washed three times (5 min per wash) in 50% ethanol, followed by decolorization with 6% H_2_O_2_ in ethanol for 24 h in the dark and incubation in a hybridization buffer (20 mmol/L Tris-HCl pH 8.0, 0.9 mol/L NaCl, 0.01% sodium dodecyl sulfate, 30% formamide) containing 10 pmol/mL of the 6-FAM-labeled *Wolbachia 16S rRNA* probe and the Cyanine3-labeled *Rickettsia 16S rRNA* probe as described previously [32]. After overnight incubation, the samples were thoroughly washed in the washing buffer (0.3 mol/L NaCl, 0.03 mol/L sodium citrate, 0.01% sodium dodecyl sulfate) and then observed under a Nikon eclipse Ti-U FluoView inverted microscope (Nikon Instruments Inc., Tokyo, Japan).

### 2.6. Data Analysis

The Bio-Rad real-time PCR system (USA) and accompanying software (Bio-Rad CFX Manager) were used for qPCR data normalization, and the relative quantities of endosymbionts were calculated using the method of 2^−ΔΔCt^ [33]. Statistical analysis was performed using IBM SPSS Statistics version 19.0 (SPSS 19.0) (IBM, Armonk, NY, USA). Tukey’s HSD was used for analysis of variance (ANOVA) to indicate significant differences between different nymphs or adults of whitefly. Figures were generated using SigmaPlot 10.0. Error bars in all graphs represent standard error.

## 3. Results

### 3.1. Rickettsia and Wolbachia Detection in Different Asia II1 Whiteflies

**Figure 1:**
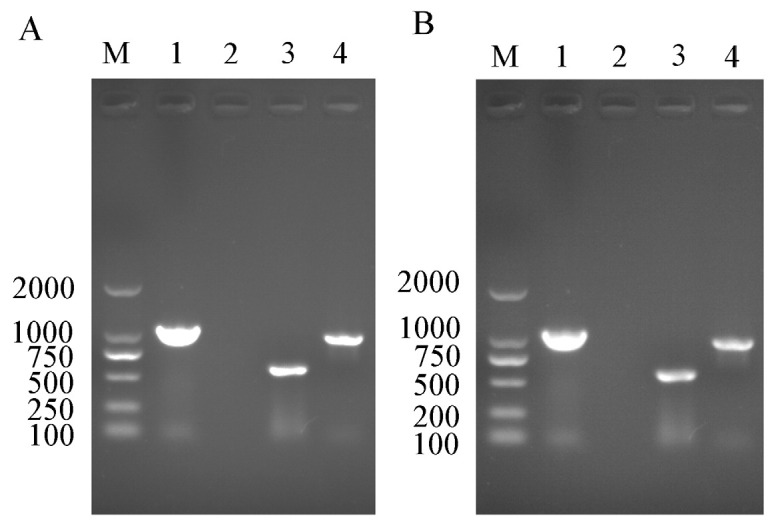
PCR detection of *Rickettsia* and *Wolbachia* in different Asia II 1 whitefly lines. (**A**,**B**): respectively, the PCR detection endosymbiont female and male in the I_WR_ whitefly population; M: DNA marker; Lanes 1: positive control (*Portiera*); Lanes 2: negative control (ddH_2_O); Lanes 3: sequence fragments of *Wolbachia wsp* gene in the I_WR_ whitefly populations; Lanes 4, sequence fragments of *Rickettsia* 16S rDNA gene in I_WR_ whitefly population.

In order to confirm the infection status of *Rickettsia* and *Wolbachia* in the I_WR_ line of *B. tabaci* Asia II1, PCR detection was confirmed by the presence of *Rickettsia* and *Wolbachia* in adult males and females of *B. tabaci* Asia II1 (Figure 1).

### 3.2. Maternal Transmission Efficiency of Rickettsia and Wolbachia of AsiaII1 I_WR_ Line

The maternal transmission efficiency of *Rickettsia* and *Wolbachia* in the I_WR_ line of Asia II1 whiteflies was detected in the arrhenotokous male offspring of virgin females, amphigenetic female offspring of I_WR_ females crossed males, indicating that the revealed complete (100%) maternal transmission of *Rickettsia* and *Wolbachia* was observed in the male and female progeny of I_WR_ line (Table 1).

### 3.3. Quantitative Detection of Rickettsia and Wolbachia in Eggs of Asia II1 Whitefly

**Figure 2:**
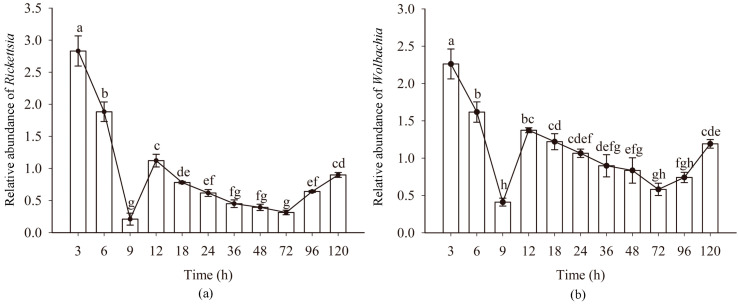
The relative abundance of *Rickettsia* and *Wolbachia* in *B. tabaci* eggs at different time intervals: (**a**) relative abundance of *Rickettsia*; (**b**) relative abundance of *Wolbachia*. *n* = 3 biological replicates. Error bars represent the standard error of the mean; Means marked with different letters are significantly different from each other (*p* ≤ 0.05).

The titers *Rickettsia* and *Wolbachia* in eggs of Asia II1 *B. tabaci* showed a consistent pattern (“W” shape) at different time intervals. The titers of endosymbionts *Rickettsia* and *Wolbachia* showed a downward trend at 3–9 h and 12–72 h, especially at 3–9 h. The titers of endosymbionts *Rickettsia* and *Wolbachia* showed an upward trend at 9–12 h and 72–120 h. Compared with other time intervals, the increase at 9–12 h was significantly higher (Figure 2). During the dynamic change of the titer of symbiotic bacteria, the titer of *Rickettsia* and *Wolbachia* was the highest in 3 h eggs and the lowest in 9 h eggs. The variation of *Protiera* titer showed the same as that of *Rickettsia* and *Wolbachia* (Figure S1).

### 3.4. FISH Visualization of Rickettsia and Wolbachia in Eggs at Different Times

**Figure 3:**
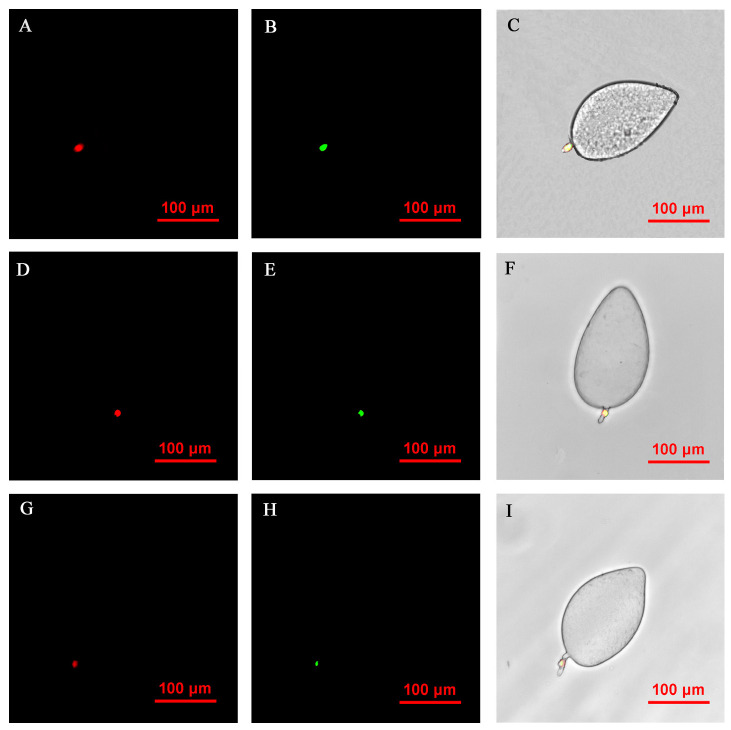
FISH visualization of *Wolbachia* and *Rickettsia* in egg stages (3–9 h) of *B. tabaci.* Localization of symbiotic bacteria *Rickettsia* (red) and *Wolbachia* (green) in the *B. tabaci* eggs at different times ((**A**–**C**): 3 h, (**D**–**F**): 6 h, (**G**–**I**): 9 h). *Rickettsia* fluorescence (**A**,**D**,**G**) and *Wolbachia* fluorescence (**B**,**E**,**H**) are displayed in dark field; W*olbachia* and *Rickettsia* fluorescence (**C**,**F**,**I**) is displayed in bright field.

**Figure 4:**
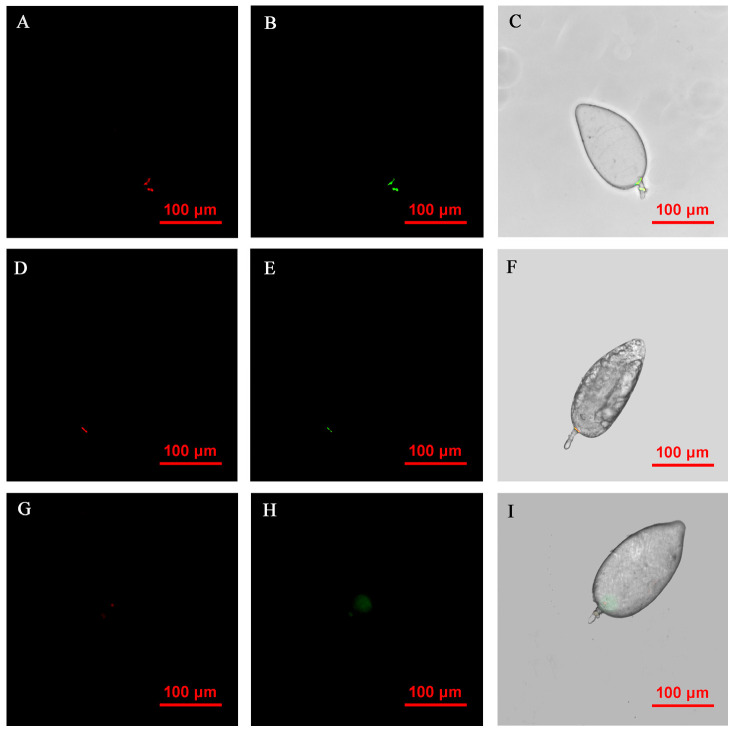
FISH visualization of *Wolbachia* and *Rickettsia* in egg stages (12–24 h) of *B. tabaci.* Localization of symbiotic bacteria *Rickettsia* (red) and *Wolbachia* (green) in the *B. tabaci* eggs at different times ((**A**–**C**): 12 h, (**D**–**F**): 18 h, (**G**–**I**): 24 h). *Rickettsia* fluorescence (**A**,**D**,**G**) and *Wolbachia* fluorescence (**B**,**E**,**H**) are displayed in dark field; W*olbachia* and *Rickettsia* fluorescence (**C**,**F**,**I**) is displayed in bright field.

**Figure 5:**
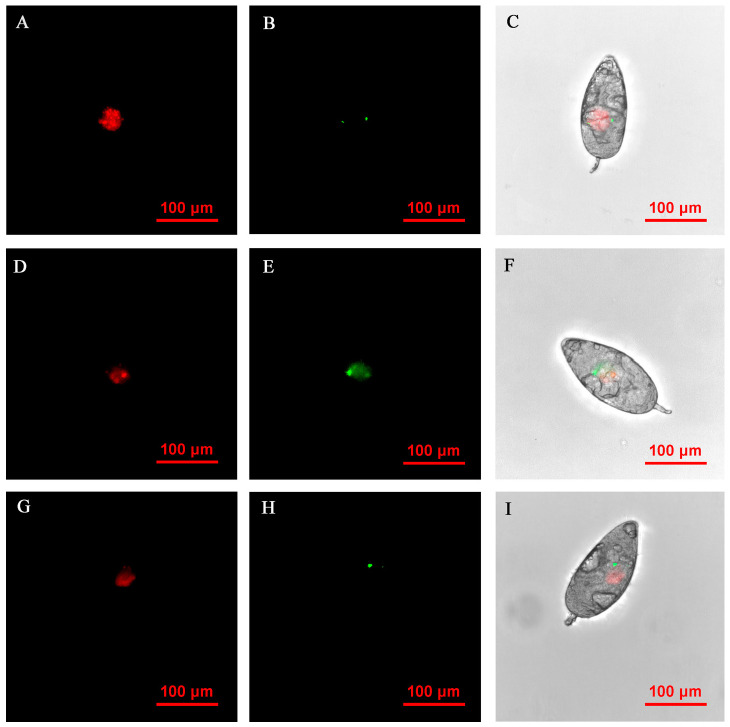
FISH visualization of *Wolbachia* and *Rickettsia* in egg stages (36–72 h) of *B. tabaci.* Localization of symbiotic bacteria *Rickettsia* (red) and *Wolbachia* (green) in the *B. tabaci* eggs at different times ((**A**–**C**): 36 h, (**D**–**F**): 48 h, (**G**–**I**): 72 h). *Rickettsia* fluorescence (**A**,**D**,**G**) and *Wolbachia* fluorescence (**B**,**E**,**H**) are displayed in dark field; W*olbachia* and *Rickettsia* fluorescence (**C**,**F**,**I**) is displayed in bright field.

**Figure 6:**
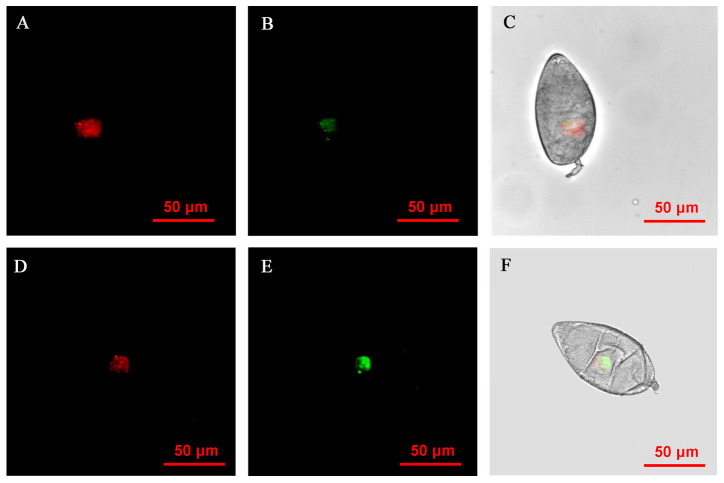
FISH visualization of *Wolbachia* and *Rickettsia* in egg stages (96–120 h) of *B. tabaci.* Localization of symbiotic bacteria *Rickettsia* (red) and *Wolbachia* (green) in the *B. tabaci* eggs at different times ((**A**–**C**): 96 h, (**D**–**F**): 120 h). *Rickettsia* fluorescence (**A**,**D**) and *Wolbachia* fluorescence (**B**,**E**) are displayed in dark field; W*olbachia* and *Rickettsia* fluorescence (**C**,**F**) is displayed in bright field.

FISH visualization showed that *Rickettsia* and *Wolbachia* in I_WR_ eggs of the Asia II1 *B. tabaci* were mainly distributed in the egg stalk position within 3–9 h, and the fluorescence signal intensity of *Rickettsia* and *Wolbachia* becomes weaker and weaker as time goes by (Figure 3). The locations of *Rickettsia* and *Wolbachia* were transferred from the egg stalk to the base of the egg, and the fluorescent signals of *Rickettsia* and *Wolbachia* in the egg stalk gradually disappeared within 12–24 h (Figure 4). *Rickettsia* and *Wolbachia* moved from egg base to the apical portion of the egg within 24–48 h (Figure 5). Finally, *Rickettsia* and *Wolbachia* moved back from the apical egg to the basal egg and were fixed in the middle of the egg to complete the transfer process (Figure 6).

### 3.5. Quantitative Detection of Rickettsia and Wolbachia in Asia II1 Whiteflies

**Figure 7:**
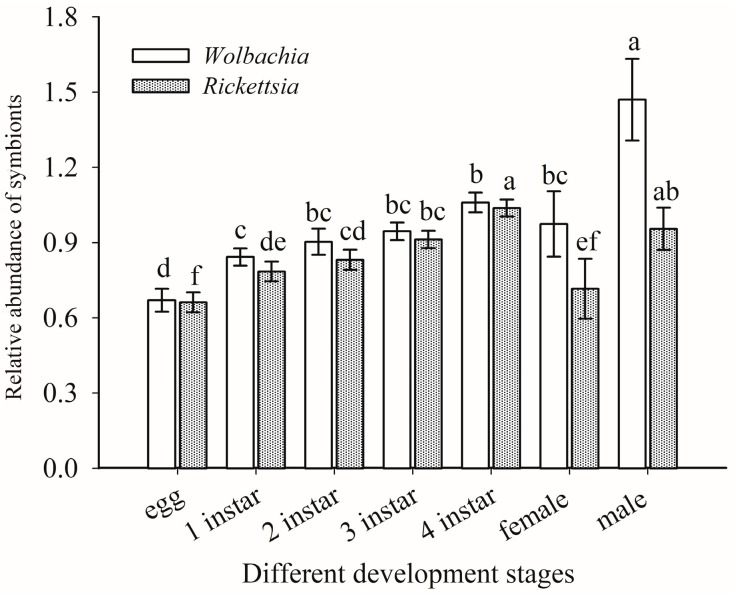
Relative abundance of *Wolbachia* and *Rickettsia* in different life stages of *B. tabaci* Asia II 1. *n* = 3 biological replicates. The column and error bars represent the fold change in mean ± SE. Means marked with different letters are significantly different from each other (*p* ≤ 0.05).

The titers of *Rickettsia* and *Wolbachia* nymphal and adult life stages of Asia II1 *B. tabaci* generally increased with the development of whiteflies. The titers of endosymbionts *Rickettsia* and *Wolbachia* in males of Asia II1 *B. tabaci* was significantly higher than those in females. The highest titer of *Wolbachia* was observed in male adults, followed by fourth instar larvae (Figure 7). The titers of *Rickettsia* in the male adults were significantly higher than *Wolbachia* (*t* = 7.816, *df* = 4, *p* = 0.001), whereas the titer of *Rickettsia* observed in nymphs were significantly higher than the titer of adult females (Figure 7).

### 3.6. FISH Visualization of Rickettsia and Wolbachia in Nymphs and Adults

**Figure 8:**
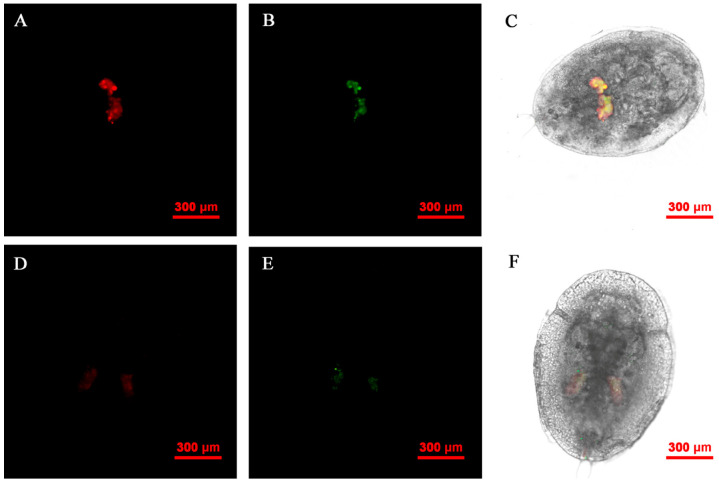
FISH visualization of *Wolbachia* and *Rickettsia* in nymph stages of *B. tabaci*. Localization of symbiotic bacteria *Rickettsia* (red) and *Wolbachia* (green) in nymph stages of *B. tabaci*. *Rickettsia* fluorescence (**A**,**D**) and *Wolbachia* fluorescence (**B**,**E**) are displayed in dark field; W*olbachia* and *Rickettsia* fluorescence (**C**,**F**) is displayed in bright field.

**Figure 9:**
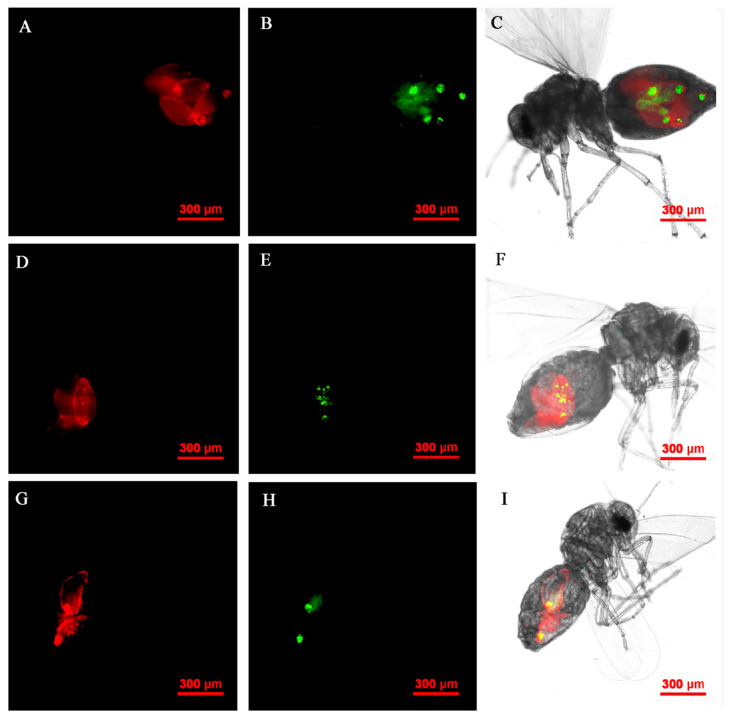
FISH visualization of *Wolbachia* and *Rickettsia* in adult stages of *B. tabaci*. Localization of symbiotic bacteria *Rickettsia* (red) and *Wolbachia* (green) in adult stages of *B. tabaci*. *Rickettsia* fluorescence (**A**,**D**,**G**) and *Wolbachia* fluorescence (**B**,**E**,**H**) are displayed in dark field; W*olbachia* and *Rickettsia* fluorescence (**C**,**F**,**I**) is displayed in bright field.

The *Rickettsia* and *Wolbachia* of Asia II1 *B. tabaci* nymph were located by FISH. The results revealed that the presence of *Rickettsia* and *Wolbachia* was limited to the bacteriocytes in the abdomen (Figure 8). *Rickettsia* and *Wolbachia* can be seen in the center of the abdomen in the adults of Asia II1 *B. tabaci*. This distribution of endosymbionts is called “the scattered pattern” (Figure 9).

## 4. Discussion

*B. tabaci* is a complex composed of at least 40 morphologically indistinguishable species that is known to harbor at least 7 endosymbionts, with infection frequencies varying between cryptic species and conspecific populations [34,35]. In this study, diagnostic PCR screening revealed that the experimental population of *B. tabaci* Asia II1 included *Rickettsia*, *Wolbachia*, *Hemipteriphilus*, and *Fritschea* (Figure S2). The species of endosymbionts infecting *B. tabaci* were affected by many factors, including climate, temperature, and geographical populations. For example, the populations of *B. tabaci* MEAM1 from Zhejiang and Beijing (China) were infected with two secondary endosymbionts (*Rickettsia* and *Hamiltonella*), whereas the populations of *B. tabaci* MEAM1 from Montenegro also contained a third endosymbiont (*Wolbachia*). The Guangzhou population of *B. tabaci* MEAM1 contains *Rickettsia*, *Hamiltonella*, and *Hemipteriphilus* [36]. The above findings suggest that host–endosymbiont interactions may be associated with environmental factors, including climate, geographical factors, and host plant [37].

The vertical transmission of endosymbionts in eggs has been recognized as the main strategy for its persistence in insect hosts, and the vertical transmission mechanism of symbiotic bacteria has been clearly studied [9]; however, the changes in the titers and location of symbiotic bacteria are still unclear. This study indicates that with the increase in egg age, *Rickettsia* and *Wolbachia* moved from the egg stalk to the egg, and the content of symbiotic bacteria (*Rickettsia* and *Wolbachia*) in eggs increased obviously for the first time at 12 h. The fluorescence intensity and the infection area of symbiotic bacteria in the FISH diagram did not reflect the change in *Rickettsia* and *Wolbachia* content at 12–24 h. The infected cell area of symbiotic bacteria increased after the symbiotic bacteria are transferred from the stalk position to the egg base, resulting in a decrease in the content of symbiotic bacteria per unit area, which is best proved by the FISH picture of the original symbiotic bacteria *Portiera* in the 1-day-old whitefly eggs and the localization of *Wolbachia* in the 1-day-old eggs of citrus psylla [38,39]. Although the ct value of the reference gene fluctuates in eggs at different times, the non-normalized data and ct-values of the reference gene were displayed in Figure S3, the ct values *coxA* and *gltA* gene were presented in Figures S4 and S5, in order to better understand the changing law of symbiotic bacteria. At the same time, the titer change of *Rickettsia* and *Wolbachia* can be reasonably explained from the biological point of view (Supplementary File 2). In this study, symbiotic bacteria *Wolbachia* and *Rickettsia* are distributed near the base of the egg stalk, which is similar to the localization of *Portiera* in the cryptophyte of *B. tabaci* MEAM1 shown by Shan et al. [38]. In addition, in the eggs of Asia II1 *B. tabaci*, only one cell composition was observed at different times from *Wolbachia* and *Rickettsia*, which is similar to the composition of symbiotic bacteria *Hamiltonella*, *Cardinium*, and *Rickettsia* observed in the eggs of different geographical populations of *B. tabaci* [26,40]. However, these results are different from the two cell structure compositions of *Hamiltonella* and *Arsenophonus* in greenhouse whitefly eggs observed by Marisa et al. [40]. These changes in cellular composition can be related to the interaction between different hosts and symbiotic bacteria.

The titers of endosymbionts *Rickettsia* and *Wolbachia* in the males of Asia II1 *B. tabaci* observed during this study were significantly higher than those in the endosymbionts of different nymphal instars and adult females. Furthermore, the titers of endosymbionts *Rickettsia* and *Wolbachia* in adult females were significantly lower than fourth instar nymphs. These results are consistent with those of Lv et al. [31], who also observed that *Wolbachia* titers in Asia II 7 *B. tabaci* males were higher than in females. The presence of higher *Wolbachia* titers in males than females can be largely related to their functions [31]. Nguyen et al. [28] revealed that the *Wolbachia* titers (capable of inducing CI) in thrips were higher in male hosts than in female hosts. The localization of *Rickettsia* and *Wolbachia* examined in nymphal instars and adults of Asia II1 *B. tabaci* by FISH revealed the presence of *Rickettsia* and *Wolbachia* in a confined pattern limited to the bacteriocytes localized in the abdomen. These results are consistent with Gottlieb et al. [32], who detected *Wolbachia* around and inside the bacteriocytes of nymphs and adults; however, in some individuals, the bacterium was also found in the abdomen.

## 5. Conclusions

In conclusion, these results will improve our understanding of the vertical transmission of symbiotic bacteria in insects and also provide basic information on the quantity and localization of *Wolbachia* and *Rickettsia* within different life stages of *B. tabaci*. These findings will serve as a reference for the interaction between symbiotic bacteria and different tissues of host insects and the horizontal transfer of symbiotics between different hosts for their management through cytoplasmic incompatibility strategy by microinjection technique.

## Figures and Tables

**Table 1:** Maternal transmission efficiency of *Rickettsia* and *Wolbachia* of Asia II1 I_WR_ line.

Whitefly Samples	*n* Females	*n* Offspring Per Female	Total *N* Tested	*Rickettsia*				*Wolbachia*	
				*n* ^+^	*n* ^−^	%	*n* ^+^	*n* ^−^	%
♀I_WR_	10	10	100	100	0	100	100	0	100
♀I_WR_ × I_WR_♂	10	10	100	100	0	100	100	0	100

*n*, numbers of; *n*^+^, the numbers of positive individuals; *n*^−^, the numbers of negative individuals.

## Data Availability

Upon request, the authors can provide the original data used in this paper.

## Supplementary Materials

The following supporting information can be downloaded at: https://www.mdpi.com/article/10.3390/insects14040401/s1, Figure S1: The endosymbionts information of Asia II1 whitefly *B. tabaci*; Figure S2: The relative quantity of *Portiera* in different times eggs of *B. tabaci*; Figure S3: Ct value of beta-actin gene in whitefly eggs at different time Error bars represent the standard error of the mean; *n*= 3 biological replicates; Figure S4: Ct value of *coxA* gene in whitefly eggs at different time Error bars represent the standard error of the mean; *n*= 3 biological replicates; Figure S5: Ct value of *gltA* gene in whitefly eggs at different time Error bars represent the standard error of the mean; *n*= 3 biological replicates; Table S1: The primers and reaction program for PCR detection of endosymbionts in Asia II1 *B. tabaci*; Table S2: The information of oligonucletide primers used in quantitative PCR in Asia II1 *B. tabaci*; Supplementary file 1: PCR detection of secondary endosymbionts in Asia II1 whitefly; Supplementary file 2: Biological explanation of dynamic change of symbiotic bacteria titer [26,27,41,42,43,44,45,46].
