# Supplementary material for: The Dynamic Distribution of Wolbachia and Rickettsia in AsiaII1 Bemisia tabaci
Source: Insects. 2023 Apr 21;14(4):401. doi: 10.3390/insects14040401 (PMC10144568; doi:10.3390/insects14040401)
Supplement: Supplementary file 1 [file insects-14-00401-s001.zip › insects-2329605-supplementary.pdf]

Table S1. The primers and reaction program for PCR detection of endosymbionts in Asia II1 *Bemisia tabaci.*

Symbiont	Primer name	Primer sequence (5′-3′)	amplicon size (bp)	Reaction program
Portiera [a]	28 F<br>1098 R	TGCAAGTCGAGCGGCATCAT<br>AAAGTTCCCGCCTTATGCGT	1000	95°C 1min; 60°C 1min, 72°C 1min (5 cycles); 95°C 1min, 58°C 1min, 72°C 1min (25 cycles); 72°C 20min
Hamiltonella [b]	Ham F<br>Ham R	TGAGTAAAGTCTGGAATCTGG<br>AGTTCAAGACCGCAACCTC	700	95°C 1min; 60°C 1min, 72°C 1min (5 cycles); 95°C 1min, 58°C 1min, 72°C 1min (30 cycles); 72°C 20min
Cardinium [c]	CFB F<br>CFB R	GCGGTGTAAAATGAGCGTG<br>ACCTMTTCTTAACTCAAGCCT	400	94°C 4min; 94°C 40s, 57°C 40s, 72°C 45s (35 cycles); 72°C 5min
Rickettsia [d]	Rb F<br>Rb R	GCTCAGAACGAACGCTATC<br>GAAGGAAAGCATCTCTGC	900	95°C 2min; 92°C 30s, 58°C 30s, 72°C 30s (30 cycles); 72°C 5min
Arsenophonus [e]	Ars23S-1<br>Ars23S-2	CGTTTGATGAATTCATAGTCAAA<br>GGTCCTCCAGTTAGTGTTACCCAA C	600	94°C 5min; 94°C 30s, 52°C 30s, 72°C 1min (35 cycles); 72°C 5min
Fritschea [f]	U23F<br>23SIGR	GATGCCTTGGCATTGATAGGCGAT GAAGGA<br>TGGCTCATCATGCAAAAGGCA	600	95°C 5min; 94°C 1min, 55°C 1min, 72°C 1.5min (35 cycles); 72°C 5min
Hemipteriphilus [g]	OLO F<br>OLO R	GCTCAGAACGAACGCTRKC<br>TTCGCCACTGGTGTTCCTC	700	94°C 4min; 94°C 30s, 60°C 1min, 72°C 2min (35 cycles); 72°C 5min
Wolbachia [h]	wsp-81F<br>wsp-691 R	TGGTCCAATAAGTGATGAAGAAAC<br>AAAAATTAAACGCTACTCCA	600	95°C 3min; 94°C 35s, 55°C 30s, 72°C 30s (40 cycles); 72°C 10min

[a] Sequences obtained from Zchori-Fein and Brown. (2002) [41].

[b] Sequences obtained from Chiel et al. (2007) [42].

[c] Sequences obtained from Weeks et al. (2003) [43].

[d] Sequences obtained from Gottlieb et al. (2006) [26].

[e] Sequences obtained from Thao and Baumann (2004) [44].

[f] Sequences obtained from Everett et al. (2005) [45].

[g] Sequences obtained from Bing et al. (2013) [46].

[h] Sequences obtained from Zhou et al. (1998) [27].

Table S2 The information of oligonucletide primers used in quantitative PCR in Asia II1 *Bemisia tabaci*

Gene	Primer name	Primer sequence	Reference
Wolbachia	Cox14F	GCCAGTATTTGGTTATATGGGAATG	[28]
coxA	Cox14R	CTCGCTAAGCCCAACAGTA	
Rickettsia	gltA-F	CGGATTGCTTTACTTAC	[30]
gltA	gltA-R	AAATACGCCACCTCTA	
β-Actin	Actin-QF	TCTTCCAGCCATCC TTCTTG	[29]
	Actin-QR	CGGTGATTTCCTTCTGCA TT	

Supplementary file 1

PCR detection of secondary endosymbionts in Asia II1 whitefly

In order to evaluation the infection species and status of endosymbionts in the Asia II1 whitefly , newly emerged adults were randomly selected in the experiment population were measured by PCR with special primers of *16S rRNA, 23S rRNA* and *wsp* genes of endosymbionts. Results revealed that the experiment population of Asia II1 whitefly was infected with one primary endosymbiont (*Portiera*) and four secondary endosymbionts (*Rickettsia, Wolbachia, Hemipteriphilus, Fritschea*), but not infected with *Cardinium* and *Hamiltonella*. The infection percentages of *Rickettsia and Hemipteriphilus in* whitefly adults were 100%, while The infection percentages of *Wolbachia* and *Fritschea were* respectively 83.3% and 46.67%, and the infection rate of males was higher than that of females.

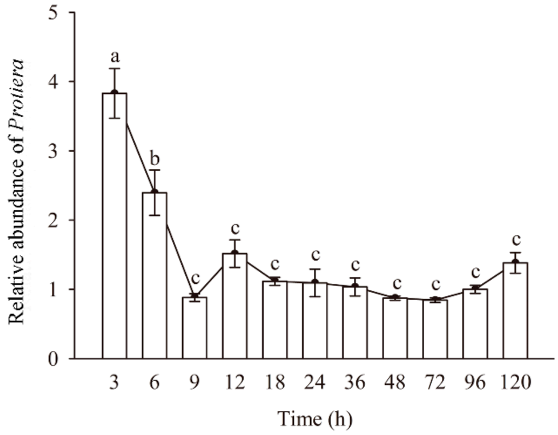

Figure S1 The relative abundance of *Portiera* in different times eggs of *B. tabaci.*

Error bars represent the standard error of the mean; Means marked with different letters are significantly different from each other ($P \leqslant 0.05$). n= 3 biological replicates. A β - actin whitefly gene was selected as an internal control for data standardization and quantification

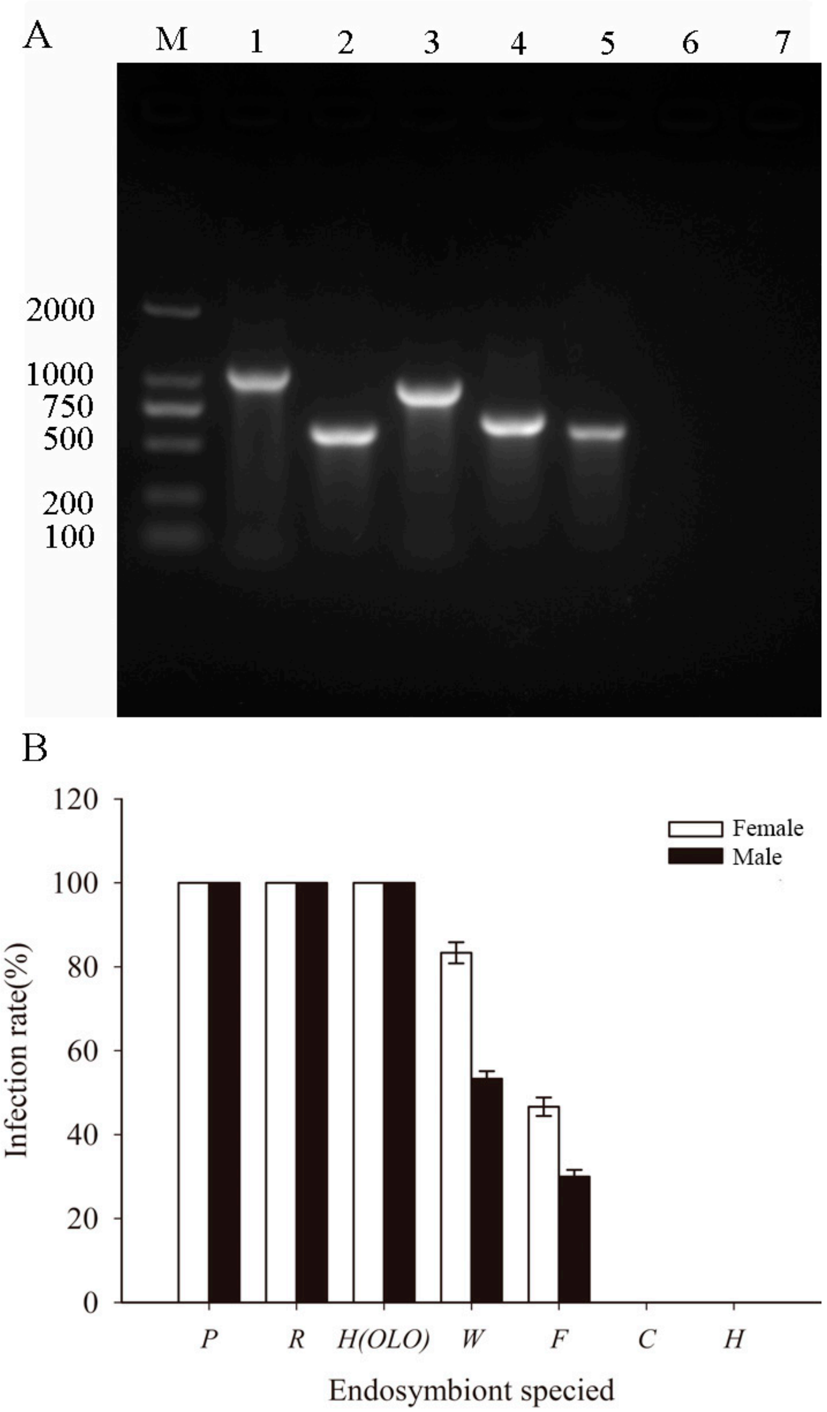

Figure S2 The endosymbionts information of Asia II 1 whitefly *B. tabaci*

Note: A: The endosymbiont species infected in whitefly, M: DNA marker; 1: *Portiera*; 2: *Wolbachia*; 3: *Rickettsia*; 4: *Hemipteriphilus*; 5: *Fritschea*; 6: *Cardinium*; 7: *Hamiltonella*. B: The endosymbionts infection rates of whitefly. *P*: *Portiera*; *R*: *Rickettsia*; *H(OLO): Hemipteriphilus*; *W*: *Wolbachia*; *F*: *Fritschea*; *C*: *Cardinium*; *H*: *Hamiltonella*. all error bars

represent standard error of mean.

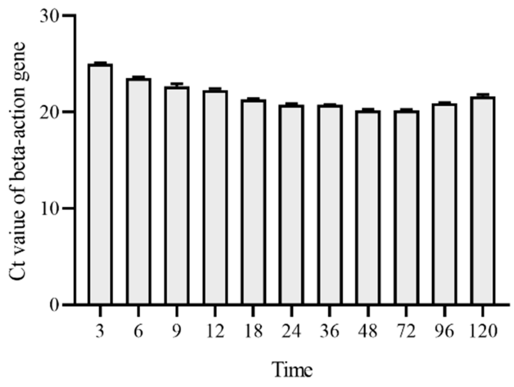

Figure S3 Ct value of beta-actin gene in whitefly eggs at different time

Error bars represent the standard error of the mean; *n*= 3 biological replicates.

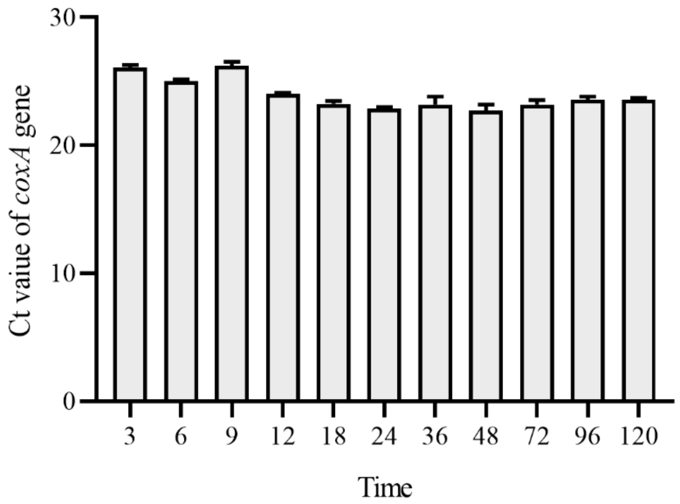

Figure S4 Ct value of *coxA* gene in whitefly eggs at different time

Error bars represent the standard error of the mean; *n*= 3 biological replicates.

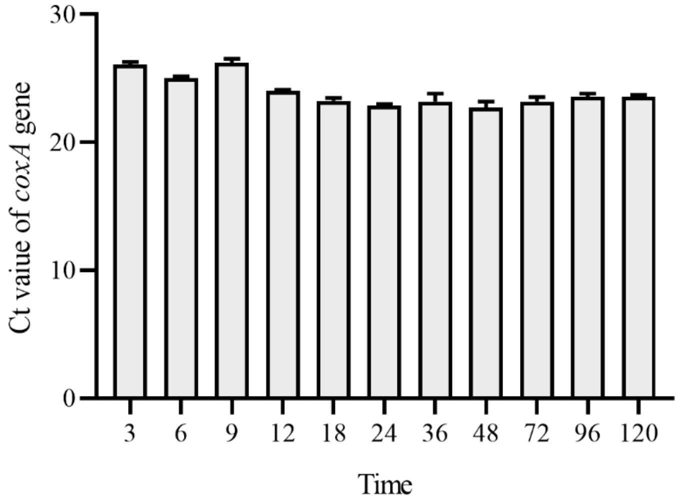

Figure S5 Ct value of *gltA* gene in whitefly eggs at different time

Error bars represent the standard error of the mean; *n*= 3 biological replicates.

Supplementary file 2

Biological explanation of dynamic change of symbiotic bacteria titer

According to the biological characteristics of symbiotic bacteria, the reasons for the dynamic change of *Rickettsia* and *Wolbachia* titers were as follows: The titers of *Rickettsia* and *Wolbachia* decreased from 3 h to 9 h, and the fluorescence intensity of *Rickettsia* and *Wolbachia* also decreased from 3 h to 9 h by FISH detection. The main reason why the titer of *Rickettsia and Wolbachia* decreased was that the egg stalk could not provide a better cell environment, which *Rickettsia* and *Wolbachia* were intracellular symbiotic bacteria. When *Rickettsia and Wolbachia* enter the egg from the egg stalk, the titer and distribution range of *Rickettsia and Wolbachia* increase, which leads to

the decrease of fluorescence intensity of *Rickettsia and Wolbachia* per unit area, at the same time, the detection sensitivity of FISH was lower than that of qPCR, therefore, FISH analysis can not support the increase of the titers of *Rickettsia and Wolbachia* within 12-18 h. The reason why the titer of *Rickettsia and Wolbachia* decreased in 12-72 h was that it was lost with the transfer of *Rickettsia and Wolbachia* in eggs. Symbiotic bacteria may infect other tissues, such as Salivary gland, fat extraction and so on, in the process of transferring in egg, *Rickettsia* and *Wolbachia* was eventually fixed in the middle of the egg, and the titers of *Rickettsia and Wolbachia* increased with time.
